# A Low Temperature Growth of Cu_2_O Thin Films as Hole Transporting Material for Perovskite Solar Cells

**DOI:** 10.3390/ma15217790

**Published:** 2022-11-04

**Authors:** Anna L. Pellegrino, Francesca Lo Presti, Emanuele Smecca, Salvatore Valastro, Giuseppe Greco, Salvatore Di Franco, Fabrizio Roccaforte, Alessandra Alberti, Graziella Malandrino

**Affiliations:** 1 Dipartimento di Scienze Chimiche, Università degli Studi di Catania, INSTM UdR Catania, Viale Andrea Doria 6, 95125 Catania, Italy; 2 National Research Council-Institute for Microelectronics and Microsystems (CNR-IMM), Zona Industriale Strada VIII No. 5, 95121 Catania, Italy

**Keywords:** HTL layer, chemical vapor deposition, hybrid perovskite

## Abstract

Copper oxide thin films have been successfully synthesized through a metal–organic chemical vapor deposition (MOCVD) approach starting from the copper bis(2,2,6,6-tetramethyl-3,5-heptanedionate), Cu(tmhd)_2_, complex. Operative conditions of fabrication strongly affect both the composition and morphologies of the copper oxide thin films. The deposition temperature has been accurately monitored in order to stabilize and to produce, selectively and reproducibly, the two phases of cuprite Cu_2_O and/or tenorite CuO. The present approach has the advantages of being industrially appealing, reliable, and fast for the production of thin films over large areas with fine control of both composition and surface uniformity. Moreover, the methylammonium lead iodide (MAPI) active layer has been successfully deposited on the ITO/Cu_2_O substrate by the Low Vacuum Proximity Space Effusion (LV-PSE) technique. X-ray diffraction (XRD), field emission scanning electron microscopy (FE-SEM), and atomic force microscopy (AFM) analyses have been used to characterize the deposited films. The optical band gap (E_g_), ranging from 1.99 to 2.41 eV, has been determined through UV-vis analysis, while the electrical measurements allowed to establish the p-type conductivity behavior of the deposited Cu_2_O thin films with resistivities from 31 to 83 Ω cm and carrier concentration in the order of 1.5–2.8 × 10^16^ cm^−3^. These results pave the way for potential applications of the present system as a hole transporting layer combined with a perovskite active layer in emergent solar cell technologies.

## 1. Introduction

Recently, copper-oxide compounds represent one of the most studied classes of semiconducting materials. The main advantages of these materials are related to the exceptional possibility of tuning the optical and electronic properties within their semiconducting behavior [1,2]. Copper oxide-based materials play a significant role in many technological applications, ranging from sensing [3] to catalysis [4], from photodetector (e.g., in combination with ZnO) [5,6] to the photo-electrochemical splitting of water [7,8]. In recent years, copper-based materials in the form of thin films have become objects of interest also in solar cell devices [9,10,11].

Indeed, together with the other classes of p-type layers applied in solar cell technology, organic materials such as PEDOT and spiro-OMeTAD [12] or 2,4,6-triarylpyridine [13], and transition metal oxides such as MoO_3_, V_2_O_5_, WO_3_, NiO and Cu_2_O have been explored as efficient hole transporting layers [14,15]. In particular, among the three most common and stable phases, Cu_2_O, Cu_4_O_3_, and CuO, named cuprite, paramelaconite and tenorite, respectively, the cuprite phase (Cu_2_O) has been intensively studied and applied as a p-type semiconducting material in solar cell devices [9,10,11,16,17]. Among the advantages of Cu_2_O with respect to the organic hole transporting layers are efficient hole collection, optical transparency, stability, a wide variety of synthetic strategies and low-cost production. Besides, Cu_2_O is environmentally stable and its main component, Cu, is earth-abundant and non-toxic. Other interesting Cu-based hole-transport materials, such as CuI or CuSCN layers, have been applied to inverted planar perovskite solar cells [18,19].

Cu_2_O as a thin film layer has been synthesized by several methods, including vapor and solution approaches. The most studied approaches are solution-phase methods [20,21], hydrothermal [22], electrodeposition [23], sputtering [24], molecular beam epitaxy [25], atomic layer deposition [26], thermal oxidation of copper [27], and chemical vapor deposition [28,29]. Among these approaches, metalorganic chemical vapor deposition (MOCVD) represents one of the most promising techniques due to its tunability processes, easy scaling up and industrial applicability. Moreover, the CVD approach offers the possibility of fine-tuning the composition of the Cu–O phases by easily altering the operating conditions and the chemical nature of the precursors [30].

Copper (I) complexes, e.g., Cu(hfa)(cod) [31], and [(cod)Cu(tfb-tfea)] [32] [hfa = 1,1,1,5,5,5-hexafluoro-2,4-pentanedionate, cod = 1,5-cyclooctadiene, and tfb-tfea = N-(4,4,4-trifluorobut-1-en-3-on)-6,6,6-trifluoroethylamine], have been applied to the deposition of Cu_2_O films, but metalorganic copper (II) compounds are also widely used as CVD precursors for the deposition of copper (I) oxide thin films, due to their thermal stability and clean decomposition step during the evaporation process [33]. Among them, the most common copper adducts are copper(II) β-diketonates, i.e., [Cu(acac)_2_], [Cu(tfa)_2_], [Cu(hfa)_2_·tmeda], and Cu(tmhd)_2_ (acac = 2,4-pentanedionate, tfa=1,1,1-trifluoro-2,4-pentanedionate, tmeda=N,N,N′,N′- tetramethylethylenediamine, tmhd= 2,2,6,6-tetramethyl-3,5-heptanedionate) [28,29,34,35,36,37].

To the best of our knowledge, only a few works in the literature report the thermal-CVD fabrication of pure Cu_2_O thin films without the use of H_2_ flow as a reducing agent at relatively low temperatures. Maruyama et al. reported the CVD fabrication of CuO films at T = 300 °C [37], Lay et al. reported the CVD fabrication of copper thin film in the range of T: 275–300 °C [38], Condorelli et al. stabilized the pure Cu_2_O phase at a deposition temperature of 300 °C from Cu(acac)_2_ precursor depending on the partial pressure of the oxygen flow [34]. Gupta et al. reported the photo-assisted MOCVD of Cu_2_O films starting from [Cu(tmhd)]_2_ complexes at a deposition temperature of 750 °C [39].

In the present work, we propose an in-depth study of the metalorganic chemical vapor deposition process for the reproducible and selective fabrication of both cuprite Cu_2_O and tenorite CuO copper oxide thin films starting from a β-diketonate copper (II) precursor, i.e., the bis(2,2,6,6-tetramethyl-3,5-heptanedionate) copper, Cu(tmhd)_2_. The MOCVD process has been tested in the temperature range of 250–400 °C, allowing the selective and reproducible fabrication of Cu_2_O on a large area at the lowest temperature of 250 °C, and a mixture of Cu_2_O–CuO or the pure CuO at higher temperatures. A sequential deposition of the methylammonium lead iodide (MAPI) layer allowed us to test copper oxide as the substrate for the vapor deposition of MAPbI_3_ film to realize the first part of a solar cell with a planar inverted structure. The present approach represents a simple, easily scalable, and industrially appealing process for the production of compact and homogeneous copper oxide films at relatively low temperatures. X-ray diffraction (XRD), field-emission scanning electron microscopy (FE-SEM), and atomic force microscopy (AFM) analyses allowed an accurate determination of the physicochemical properties of the deposited layers, while Hall Effect measurements enabled verifying the p-type conductivity of the deposited films. Finally, UV-vis spectra have been carried out for the determination of the optical band gap.

## 2. Materials and Methods

### 2.1. Cu_2_O Synthesis

The Cu(tmhd)_2_ compound was purchased from Sigma-Aldrich and used without further purification. The depositions were performed in a horizontal, hot-wall reactor under reduced pressure, using argon (150 sccm) as a carrier gas, and oxygen (200 sccm) as a reacting gas. The Ar and O_2_ flows were controlled using MKS 1160 flow controller units and were introduced in proximity to the reaction zone. The vacuum inside the reactor was maintained through a scroll pump unit and monitored at the value of 4 Torr using MKS Baratron 122AAX. The films were deposited on Si (001) and quartz/ITO substrates in the 250–400 °C temperature range. The precursor source was kept at 130–140 °C for an efficient vaporization process. Each section was heated independently, with ±2 °C accuracy, using K-type thermocouples and computer-controlled hardware.

### 2.2. MAPI Deposition

The MAPbI_3_ films were deposited by a Low Vacuum Proximity Space Effusion (LV-PSE) technique with specifically customized vacuum deposition equipment, provided by Kenosistec s.r.l. Lead iodide powders (99.99% purity) were purchased from Sigma Aldrich (St. Louis, MO, USA). Methyl ammonium iodide was purchased from Dyenamo AB. All materials were used as received without any further purification. The LV-PSE technique consists of a sequential deposition of PbI_2_ and MAI via physical sublimation from powders at a base pressure of ~2 × 10^−2^ mbar with the crucibles taken at 350 °C and 135 °C, respectively. The substrate was posed at a medium-range distance with respect to the sources (~2 cm). During the first step, PbI_2_ was deposited, then the conversion into MAPbI_3_ occurred through an adsorption-incorporation-migration mechanism fully described in previous works [40,41,42].

### 2.3. Characterization

Structural characterization was performed using a Smartlab Rigaku diffractometer in grazing incident mode (0.5°) operating at 45 kV and 200 mA equipped with a rotating anode of Cu K_α_ radiation. Film morphologies were investigated using field emission scanning electron microscopy (FE-SEM) ZEISS SUPRA 55 VP. The films deposited on glass were Au-coated before FE-SEM characterization. Topographic characterization was performed through Atomic Force Microscopy (AFM) adopting an Au-coated silicon probe with a nominal 35 nm tip curvature radius and a typical force constant of 0.1 N. The AFM images were obtained in contact mode. Before and after each measurement the noise level was 0.01 nm. The UV-Visible absorption spectra were recorded using a Jasco V-650 spectrophotometer. The spectra were recorded in the wavelength range from 250 to 700 nm for Cu_2_O thin films deposited on ITO-quartz substrates. Electrical characterization of the material was carried out by Hall effect measurements at room temperature using MMR H50 equipment. For this purpose, Van der Paw structures were fabricated, defining Cu/Au Ohmic contacts at the four corners of 1 cm × 1 cm Cu_2_O samples grown on a SiO_2_ substrate.

## 3. Results

An MOCVD approach has been successfully applied to the synthesis of copper oxide in form of a thin film starting from Cu(tmhd)_2_ complex. All the depositions allow the fabrication of compact and homogeneous thin films in the area of 2 cm × 2 cm on Si and quartz/ITO substrates. The effect of deposition temperature has been accurately studied in terms of both phase composition and morphology structures.

**Figure 1:**
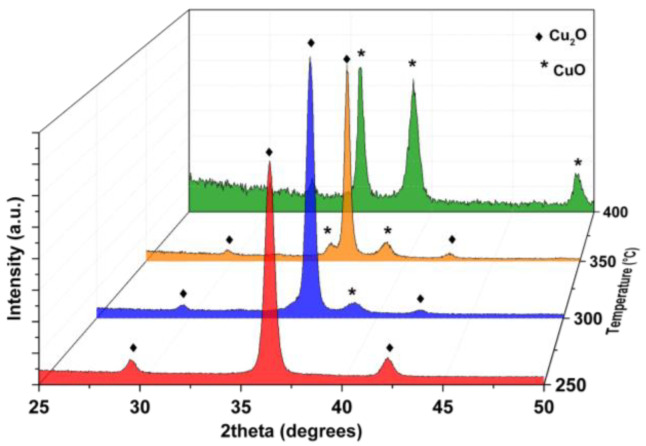
X-ray diffraction patterns of the Cu_2_O and CuO thin films prepared by MOCVD on the Si substrate at different temperatures from 250 °C (red line), 300 °C (blue line), 350 °C (orange line) to 400 °C (green line).

A complete overview of the X-ray diffraction (XRD) analysis of copper oxide thin films deposited by MOCVD in the 250–400 °C temperature range is reported in Figure 1. At the lowest temperature of 250 °C, the pattern (red line) exhibits peaks at 29.64°, 36.50° and 42.43°, which can be assigned to the 110, 111 and 200 reflections of the pure, polycrystalline cuprite phase (PDF card n. 077-0199). At a deposition temperature of 300 °C, the pattern (blue line) presents an additional peak at 38.89°, which can be ascribed to 111/200 reflections of CuO traces. In fact, at higher temperatures (350 °C—orange line) together with the previous one, the additional peak at 35.58° points to the formation of a mixture of Cu_2_O and CuO phases. Finally, at 400 °C, the pattern in green shows the formation of the pure tenorite phase CuO, with the presence of signals at 35.57°, 38.92° and 48.80° related to the 002, 111/200, and -202 reflections, respectively (PDF card n. 00-045-0937), while no peaks related to Cu_2_O are present.

The observed trend indicates a strong effect of the deposition temperature in the selective formation of Cu(I) and Cu(II) oxides, with the lowest temperature (250 °C) stabilizing the Cu_2_O phase and the highest temperature (400 °C) stabilizing the CuO one. At even lower temperatures of 200 °C, only very small, isolated nuclei are found (Figure S1a), thus indicating that 250 °C is the lowest temperature to produce significant precursor decomposition. This finding is also supported by the EDX data (Figure S1b), which do not show any Cu peak; the Cu amount is lower than the detection limit of the technique.

The Cu(I) and/or Cu(II) oxide formation can be rationalized considering a balance between two aspects: (i) the decomposition mechanism of the Cu(tmhd)_2_ precursor in the reactor, which involves the organic component of the complex acting as a reducing agent; (ii) the oxidant atmosphere under the present deposition conditions [29]. Therefore, at lower temperatures, the first aspect is prevalent and tends to stabilize the Cu(I) oxide phase due to the reducing action of the organic component; on the contrary, at higher temperatures, the second aspect prevails, resulting in the mere formation of Cu(II) oxide.

Differing from the present findings, Gupta et al. [39] reported the fabrication of Cu_2_O films from the same Cu(tmhd)_2_ precursor at a much higher deposition temperature of 750 °C, while in present experiments at a temperature of 300 °C the Cu(I) already starts to oxidize to Cu(II) and at 400 °C pure CuO forms. The reason why Gupta et al. stabilized the Cu_2_O at higher temperatures is that in addition to N_2_, used in their study as a carrier gas, a mixture of N_2_O and oxygen was used during deposition. The N_2_O was acting as a reducing agent in regard to the Cu(II), which would have stabilized for the effect of temperature. In our experiments, starting from a Cu(II) precursor, we succeeded in growing highly homogeneous Cu_2_O layers at very low temperatures, with the tmhd organic ligand acting as a reducing agent. Finally, the nature of the films deposited by Gupta et al. [39] had a reported thickness of only 15 nm and TEM observed islands in the order of 100–200 nm.

It is worth noting that in the literature only a few works report the stabilization of the cuprite crystalline phase at low temperatures, with the lowest reported temperature of 300 °C for vapor deposition approaches [28,29,32,33]. In the present case, the formation of pure Cu_2_O films has been achieved at even lower temperatures and actually, the thermal budget related to a 50 °C difference is a great advantage. In fact, the present optimized process, using a deposition temperature as low as 250 °C, paves the way for a wide application of the current Cu_2_O synthetic approach also on a variety of polymeric substrates.

**Figure 2:**
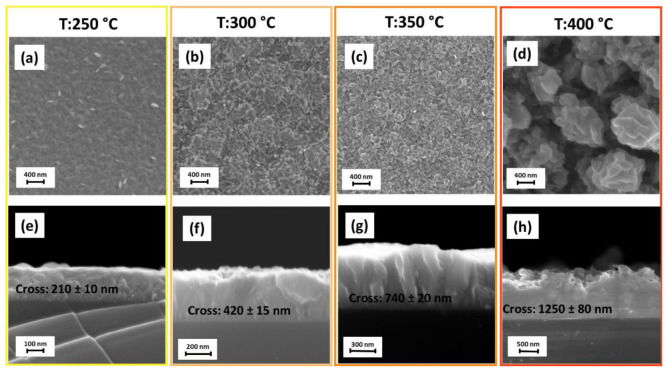
FE-SEM images of Cu_2_O and CuO thin films prepared by MOCVD on Si substrate deposited at 250 °C: (**a**) plan view and (**e**) cross-section; at 300 °C: (**b**) plan view and (**f**) cross-section; at 350 °C: (**c**) plan view and (**g**) cross-section; at 400 °C: (**d**) plan view and (**h**) cross-section.

**Figure 3:**
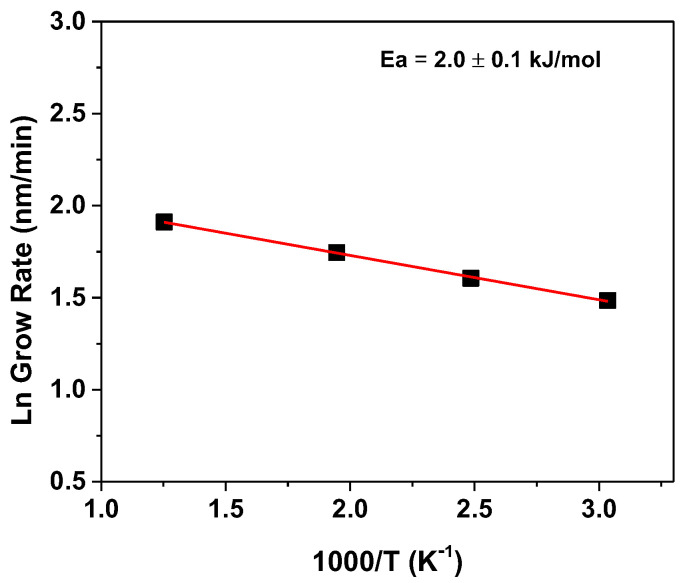
Arrhenius plot of the dependence of the logarithm of growth rate vs. 1000/T. Solid line is the linear best fit.

Morphology features of CuO and Cu_2_O thin films have been studied by field emission scanning electron microscopy (FE-SEM). The FE-SEM image of the Cu_2_O films deposited at 250 °C on Si (100) (Figure 2a) shows the formation of a very compact and homogeneous layer in which, probably due to the low fabrication temperature, a nanostructured feature is barely visible. Indeed, at higher deposition temperatures, i.e., 300 °C and 350 °C in Figure 2b,c, respectively, the films show a porous structure, probably caused by the coalescence of small grains of the order of tens of nanometers during the film growth. Finally, at 400 °C, the morphology appears much more nanostructured with the formation of plate-like grains of the order of 200–400 nm assembled into column-like structures (Figure 2d). The evident change in morphologies as a function of fabrication temperatures may be attributed on the one hand to the different contributions of nucleation and growth processes, on the other hand to the different Cu–O crystalline phase arrangements. The cross-section images display a growth trend, with an increase from 220 nm to 420 nm at 250 °C and 300 °C (Figure 2e,f), and up to 740 nm for the layer obtained at 350 °C (Figure 2g). The film deposited at 400 °C shows, indeed, a thicker and rugged profile, with a thickness of about 1250 nm (Figure 2h). Specifically, growth rates vary from 3, 7, 12 and 21 nm/min for 250, 300, 350 and 400 °C, respectively. To explain the dependence of the growth rate as a function of temperature, the Arrhenius plot (Figure 3) has been derived, even though the formed Cu–O phases differ in the investigated temperature range.

Despite the increase in thickness variation with increasing temperature, the growth rate dependence does not suggest the presence of a kinetic regime and points to a mass transport-limited regime. This evidence is supported by the derived low activation energy, obtained from the slope of the linear fitting analysis, equal to 2.0 ± 0.1 kJ/mol.

Hence, optical absorptions have been evaluated in order to determine the band gap of the semiconductor Cu_2_O thin films. Particularly, UV-vis spectra have been measured for Cu_2_O thin films of various thicknesses, deposited on ITO/quartz substrate.

With the aim of applications as a hole transporting layer (HTL) in solar cell devices, the thickness of the Cu_2_O films has been finely tuned and optimized. Therefore, different depositions have been carried out at 250 °C and with different vaporization temperatures and deposition times, i.e., (I) T_vap_: 140 °C, t: 30 min; (II) T_vap_: 140 °C, t: 15 min and (III) T_vap_: 130 °C t: 15 min, in order to have different-thickness Cu_2_O layers. In particular, under these conditions, three samples have been obtained with thicknesses of 230, 140, and 90 nm, respectively. The cross-section and the morphologies are reported in Figure S2.

**Figure 4:**
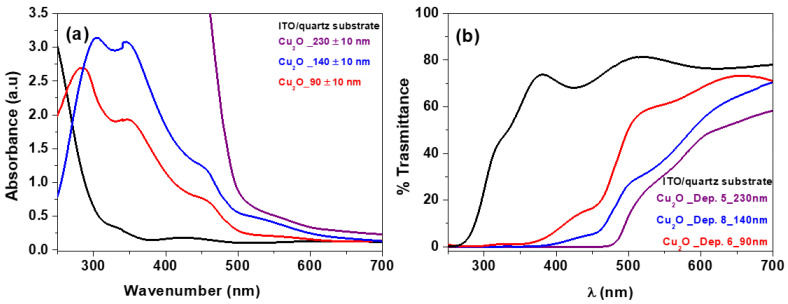
UV-vis absorbance (**a**) and transmittance (**b**) spectra of the ITO/quartz substrate (black line); Cu_2_O on ITO/quartz with thickness of 230 nm (purple line); 140 nm (blue line) and 90 nm (red line).

The absorbance spectra in the UV-vis range of the ITO/quartz substrate and the Cu_2_O_230 nm, Cu_2_O_140 nm and Cu_2_O_90 nm films are displayed in Figure 4a. The absorbance spectra show high transparency up to 300 nm for the ITO substrate, and an absorption edge localized between 450 and 500 nm for the Cu_2_O films, which corresponds to the excitonic band gap of Cu_2_O films. The spectra of the Cu_2_O_230 nm, Cu_2_O_140 nm and Cu_2_O_90 nm films display an increase in absorbance as a consequence of the thickness increase, a behavior already observed for films grown with different techniques [43]. Transmission spectra of ITO/quartz and Cu_2_O thin films are reported in Figure 4b, while in Figure S3 the transmission of the ITO/quartz annealed at 250 °C is compared to the untreated ITO/quartz substrate. Regardless of thickness, all Cu_2_O films absorb strongly in the UV-vis region between 250 and 450 nm, resulting in a drop in the percentage of transmission. As the thickness of the hole transporting layer is significantly reduced, the transmittance in the visible range increases. In fact, up to λ = 480 nm, the transmittance for the thickest Cu_2_O_230 nm and Cu_2_O_140 nm is minimal and then begins to rise, reaching only 20% for the Cu_2_O_230 nm and 40% for the intermediate Cu_2_O_140 nm at λ = 500 nm. Cu_2_O_90 nm thin film, on the other hand, achieves acceptable transmittance levels (about 60%) at λ = 450 nm and reaches a higher 70% at λ= 650 nm. The present results agree with the literature data [44,45], and point to the potential applicability of the presently grown Cu_2_O film as an HTL layer for photovoltaic applications [46].

**Figure 5:**
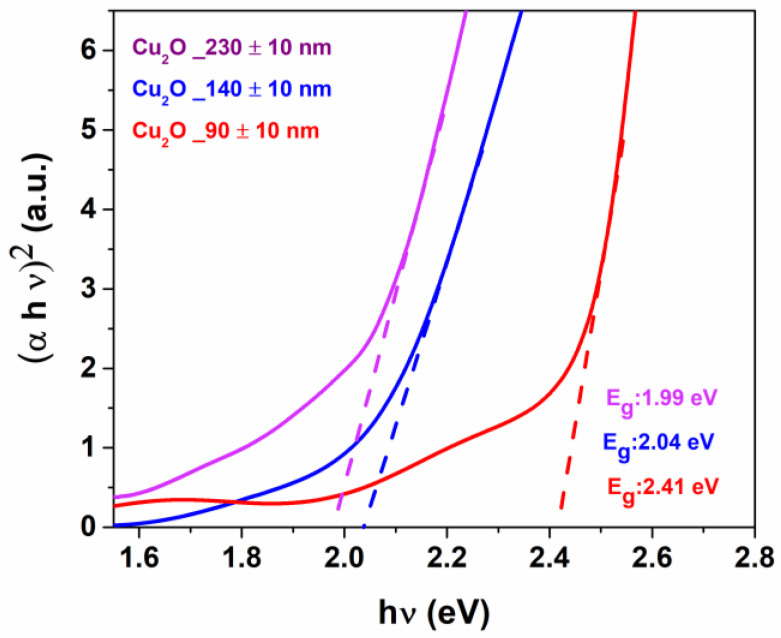
Tauc’s plot [(αhν)^2^ against photon energy (hν)] of Cu_2_O films on ITO_quartz with thickness of 230 nm (purple line); 140 nm (blue line) and 90 nm (red line).

Tauc’s plot obtained from the relation of (αhν)^2^ versus (hν) is calculated from the optical measurements reported in Figure S4 for the ITO/quartz substrate and in Figure 5 for the Cu_2_O films. Using a linear extrapolation, the values of the direct optical band gap of the Cu_2_O_230 nm, Cu_2_O_140 nm and Cu_2_O_90 nm films are evaluated to be 1.99, 2.04 and 2.41 eV, respectively. In order to evaluate the reproducibility of the process and to have statistically significant results, the band gaps from three different samples for each deposition condition of the films Cu_2_O_230 nm, Cu_2_O_140 nm and Cu_2_O_90 nm have been extrapolated yielding average values of 1.97 ± 0.02, 2.03 ± 0.03 and 2.37 ± 0.06 eV, respectively. It is interesting to note that the band gap increases upon decreasing film thickness. This band gap spreading effect has been previously observed for Cu_2_O films [47] and in similar thin film studies [48,49].

These values compare well with literature data of Cu_2_O samples deposited through spatial atomic layer deposition [31,50,51].

In order to determine the type of conductivity of the deposited Cu_2_O films, Van der Pauw and Hall Effect measurements have been performed at room temperature.

**Figure 6:**
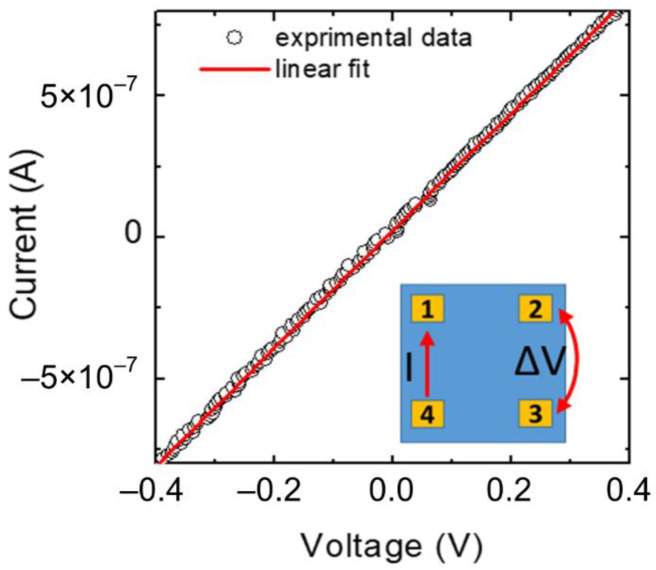
Typical current-voltage (I–V) measurement carried out on a Van der Pauw structure fabricated on Cu_2_O sample. The inset schematically illustrates the geometry on the sample and the electrical I-V measurement setup.

As an example, Figure 6 shows a typical current-voltage (I-V) measurement carried out on a Van der Pauw structure fabricated on the Cu_2_O sample. The inset in Figure 6 shows schematically the geometry of the sample and the I-V measurement setup. From the linear fit of the experimental data, the sheet resistance R_SH_ of the Cu_2_O films has been determined.

Thereafter, Hall effect measurements have been performed by applying a magnetic field of 0.1 T perpendicular to the sample surface. These measurements allowed us to determine the type of carriers responsible for the conduction and their concentration [52].

Then, combining the values of the sheet resistance, determined through the Van der Pauw measurements, with the carrier density, extracted under the application of the magnetic field, the carrier mobility has been determined.

Noteworthy, from the sign of the Hall voltage it can be concluded that all the deposited films are p-type. Moreover, no significant differences in the electrical properties have been observed by varying the Cu_2_O film thickness from 90 nm to 230 nm.

The hole concentration *p* is in the order of 1.5–2.8 × 10^16^ cm^−3^, while the values of the Hall mobility are in the range 4–7 cm^2^V^−1^s^−1^. Table 1 summarizes the main parameters extracted by the Van der Pauw and Hall effect measurements. The values reported in the table have an error of about ±20% arising from the average of different measurements acquired on the Van der Pauw structure in the different orientations.

These differences are likely due to the high contact resistance of the metal pads at the four corners of the Van der Pauw structures, but a slight non-uniformity in the film’s electrical properties cannot be excluded.

The observed values are comparable to or even better than literature data which give resistivity in the range of 20–10^3^ Ω cm [53,54] and carrier concentrations of 10^15^–10^18^ cm^−3^ [53,54].

Afterward, pure cuprite thin films are deposited on a conductive layer, i.e., ITO on a quartz substrate, starting from the optimized MOCVD parameter conditions. The Cu_2_O_ITO_quartz system has been used for the sequential deposition of the MAPI active layer as part of the stack of a solar cell with a planar inverted architecture [13,40,55,56]. An accurate study has been conducted for each step of the multilayer MAPI_Cu_2_O_ITO assembly. The system has been grown on a surface of 2 cm × 2 cm.

**Figure 7:**
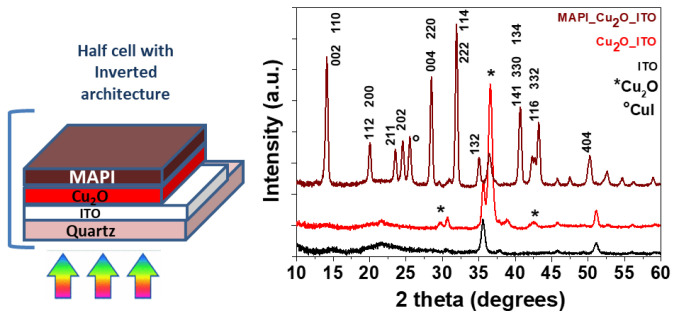
X-ray diffraction patterns of ITO substrate (black line), Cu_2_O thin film prepared by MOCVD on the ITO substrate at 250 °C (red line) and the multilayer system MAPI/Cu_2_O/ITO substrate (brown line). The * indicates the Cu_2_O peaks. On the left, a cartoon is shown of the planar inverted architecture realized up to the MAPI deposition.

X-ray diffraction patterns of the different layers have been reported in Figure 7. The pattern in brown, related to the MAPI/Cu_2_O/ITO system, displays several peaks pointing to the formation of a crystalline multilayer and associated with: (i) the pure Cu_2_O phase; (ii) the MAPI reflection peaks attributed through comparison with literature data [57]; (iii) CuI impurities. No peaks associated with the ITO substrate are detected due to the thickness of the MAPI_Cu_2_O layers. The detection of the CuI phase is likely a result of some interaction of the MAPI layer with the Cu_2_O thin film. This phenomenon is supported by the study of the interaction between the MAPI and the Cu electrode, which has been demonstrated to produce CuI [58].

**Figure 8:**
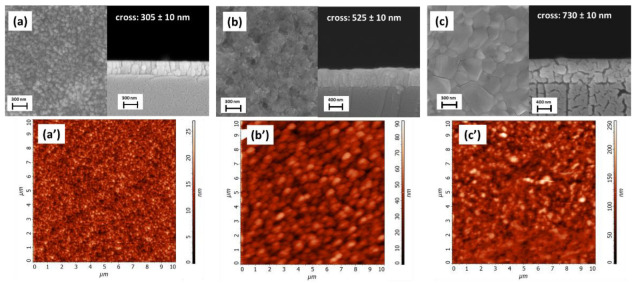
FE-SEM plan view and cross-section images of (**a**) ITO_quartz substrate; (**b**) Cu_2_O thin film deposited at 250 °C on ITO substrate; (**c**) MAPI film deposited on Cu_2_O_ITO_quartz substrate. The corresponding 10 μm × 10 μm AFM topographical images (**a’**–**c’**) are also shown.

FE-SEM images in Figure 8 show the different morphologies of the multilayer structure from the ITO substrate (Figure 8a) and the Cu_2_O film (Figure 8b) to the MAPI layer (Figure 8c). The ITO substrate is a compact homogeneous nanostructured layer with regular grains of about 50 nm and a thickness of about 305 nm. The Cu_2_O thin film, deposited on top of the ITO substrate, displays a flat morphology (Figure 8b) similar to the one found for the Cu_2_O film grown on Si (see Figure 2a), with a whole thickness of 525 ± 10 nm. This thickness, considering the previous estimation of 305 nm for the ITO, leads to a net Cu_2_O layer of about 220 ± 10 nm.

Finally, the MAPI film in Figure 8c shows a nanostructured compact surface with smooth grains of hundreds of nanometers in size. The estimated thickness of the MAPI layer is around 275 ± 10 nm, derived from the difference in the cumulative thickness of the ITO_Cu_2_O_MAPI system of 800 ± 10 nm.

Finally, the atomic force microscopy (AFM) characterization of the three layers (Figure 8a’–c’) confirms the homogeneity of the films as well with fully coalesced grains on a larger area of 10 μm × 10 µm, and a root mean square (RMS) roughness of about 2.9 nm, 8.9 nm and 26.6 nm (measured on areas of 4 μm × 4 μm) for ITO, Cu_2_O and MAPI films, respectively. The RMS roughness values of three different Cu_2_O samples, deposited under the same conditions of the Cu_2_O_230 nm, range from 7.8 to 10.1 nm. The low RMS roughness of the Cu_2_O layer is comparable to literature data on films grown on Si, further confirming these films as suited for the growth of the perovskite layer.

## 4. Conclusions

In summary, a simple approach has been optimized to produce a stack composed of MAPI/Cu_2_O/ITO/quartz. The extremely tunable MOCVD process allows the selective and reproducible fabrication of pure, uniform and highly compact Cu_2_O films on various substrates. In addition, the very low deposition temperature of the Cu_2_O layer makes this process appealing for deposition on temperature-sensitive substrates, such as polymers. In fact, the low operating temperature is a crucial issue and the capability to operate at 250 °C, using a process that is already industrially applied on a large scale, represents a breakthrough for the production of solar cells on plastic flexible supports. The electrical measurements and band gap values confirm the potentiality of the Cu_2_O layers as p-type semiconducting materials for solar cell devices. Finally, preliminary studies have been carried out to produce the MAPI/Cu_2_O/ITO stack, allowing us to scrutinize problematic issues regarding the MAPI/Cu_2_O interface.

## Figures and Tables

**Table 1:** Summary of the main parameters extracted by Van der Pauw and Hall effect measurements at room temperature.

Sample	Carrier Type	Resistivity (Ω cm)	Carrier Concentration (cm^−3^)	Mobility (cm^2^ V^−1^ s^−1^)
230 nm	Holes	2	1.5 × 10^16^	5.2
140 nm	Holes	73	2.0 × 10^16^	4.3
90 nm	Holes	32	2.8 × 10^16^	7.0

## Data Availability

Additional data are reported in the Supplementary Materials.

## Supplementary Materials

The following supporting information can be downloaded at: https://www.mdpi.com/article/10.3390/ma15217790/s1, Figure S1: FE-SEM plan-image and EDX spectrum of the Cu_2_O film deposited at 200 °C; Figure S2: FE-SEM images in plain and cross of Cu_2_O thin films; Figure S3: Transmittance spectra of the ITO/quartz substrate; Figure S4: Tauc’s plot of the ITO/quartz substrate.

## References

[1] Al-Jawhari H.A. (2015). A review of recent advances in transparent p-type Cu_2_O-based thin film transistors. Mater. Sci. Semicond. Process..

[2] Meyer B.K., Polity A., Reppin D., Becker M., Hering P., Klar P.J., Sander T., Reindl C., Benz J., Eickhoff M. (2012). Binary copper oxide semiconductors: From materials towards devices. Phys. Status Solidi B.

[3] Lupan O., Cretu V., Postica V., Ababii N., Polonskyi O., Kaidas V., Schütt F., Mishra S.K., Monaico E., Tiginyanu I. (2016). Enhanced ethanol vapour sensing performances of copper oxide. Sens. Actuators B.

[4] Mohammed A.M., Mohtar S.S., Aziz F., Mhamad S.A., Aziz M. (2021). Review of various strategies to boost the photocatalytic activity of the cuprous oxide-based photocatalyst. J. Environ. Chem. Eng..

[5] Elfadill N.G., Hashim M.R., Saron K.M.A., Chahrour K.M., Qaeed M.A., Bououdina M. (2015). Ultraviolet Visible photo-response of p-Cu_2_O/n-ZnO heterojunction prepared on flexible (PET) substrate. Mater. Chem. Phys..

[6] Ghamgosar P., Rigoni F., Shujie You S., Dobryden I., Kohan M.G., Pellegrino A.L., Concina I., Almqvist N., Malandrino G., Vomiero A. (2018). ZnO-Cu_2_O core-shell nanowires as stable and fast response photodetectors. Nano Energy.

[7] Jun Seo Y., Arunachalam M., Ahn K.-S., Hyung Kang S. (2020). Integrating heteromixtured Cu_2_O/CuO photocathode interface through a hydrogen treatment for photoelectrochemical hydrogen evolution reaction. Appl. Surf. Sci..

[8] Li C., Fang T., Hu H., Wang Y., Liu X., Zhou S., Fu J., Wang W. (2021). Synthesis and enhanced bias-free photoelectrochemical water-splitting activity of ferroelectric BaTiO_3_/Cu_2_O heterostructures under solar light irradiation. Ceram. Int..

[9] Masudy-Panah S., Zhuka S., Tana H.R., Gong X., Dalapati G.K. (2018). Palladium nanostructure incorporated cupric oxide thin film with strong optical absorption, compatible charge collection and low recombination loss for low cost solar cell applications. Nano Energy.

[10] Nguyen V.S., Sekkat A., Bellet D., Chichignoud G., Kaminski-Cachopo A., Muñoz-Rojas D., Favre W. (2021). Open-air, low-temperature deposition of phase pure Cu_2_O thin films as efficient hole-transporting layers for silicon heterojunction solar cells. J. Mater. Chem. A.

[11] Kim S., Jung Y.S., Hong J.S., Kim K.H. (2018). Fabrication of copper oxide-based heterojunction thin film solar cells using sputtering. Mol. Cryst. Liq. Cryst..

[12] Calil L., Kazim S., Gratzel M., Kurias S.A. (2016). Hole-Transport Materials for Perovskite Solar Cells. Angew. Chem. Int. Ed..

[13] Duan L., Chen Y., Jia J., Zong X., Sun Z., Wu Q., Xue S. (2020). Dopant-Free Hole-Transport Materials Based on 2,4,6-Triarylpyridine for Inverted Planar Perovskite Solar Cells. ACS Appl. Energy Mater..

[14] Messmer C., Bivour M., Schön J., Hermle M. (2018). Requirements for Efficient Hole Extraction in Transition Metal Oxide-Based Silicon Heterojunction Solar Cells. J. Appl. Phys..

[15] Chen W., Wu Y., Yue Y., Liu J., Zhang W., Yang X., Chen H., Bi E., Ashraful I., Grätzel M. (2015). Efficient and stable large-area perovskite solar cells with inorganic charge extraction layers. Science.

[16] Markose K., Shaji M., Bhatia S., Nair P.R., Saji K.J., Antony A., Jayaraj M.K. (2020). Novel boron doped p-type Cu_2_O thin film as hole selective contact in c-Si solar cell. ACS Appl. Mater. Interfaces.

[17] Jayathilaka C., Kumara L.S.R., Ohara K., Song C., Kohara S., Sakata O., Siripala W., Jayanetti S. (2020). Enhancement of Solar Cell Performance of Electrodeposited Ti/n-Cu_2_O/p-Cu_2_O/Au Homojunction Solar Cells by Interface and Surface Modification. Crystals.

[18] Khadka D.B., Shirai Y., Yanagida M., Miyano K. (2020). Ammoniated aqueous precursor ink processed copper iodide as hole transport layer for inverted planar perovskite solar cells. Sol. Energy Mater. Sol. Cells.

[19] Ye S., Sun W., Li Y., Yan W., Peng H., Bian Z., Liu Z., Huang C. (2015). CuSCN-Based Inverted Planar Perovskite Solar Cell with an Average PCE of 15.6%. Nano Lett..

[20] Nitta R., Kubota Y., Kishi T., Yano T., Matsushita N. (2020). One-step direct fabrication of phase-pure Cu_2_O films via the spin-spray technique using a mixed alkaline solution. Mater. Chem. Phys..

[21] Karle S., Rogalla D., Ludwig A., Becker H.-W., Wieck A.D., Grafen M., Devi A. (2017). Synthesis and evaluation of new copper ketoiminate precursors for a facile and additive-free solution-based approach to nanoscale copper oxide thin films. Dalton Trans..

[22] Ibupoto Z.H., Khun K., Lu J., Willander M. (2013). The synthesis of CuO nanoleaves, structural characterization, and their glucose sensing application. Appl. Phys. Lett..

[23] Borkar R., Dahake R., Rayalu S., Bansiwal A. (2018). Copper Oxide Nanograss for Efficient and Stable Photoelectrochemical Hydrogen Production by Water Splitting. J. Electron. Mater..

[24] Lakshmanan A., Alex Z.C., Meher S.R. (2022). Cu_2_O thin films grown by magnetron sputtering as solar cell absorber layers. Mater. Sci. Semicond. Process..

[25] Huo W., Shi J., Mei Z., Liu L., Li J., Gu L., Du Z., Xue Q. (2015). High-index Cu_2_O (113) film on faceted MgO (110) by molecular beam epitaxy. J. Cryst. Growth.

[26] Hu X., Schuster J., Schulz S.E., Gessner T. (2015). Surface chemistry of copper metal and copper oxide atomic layer deposition from copper(II) acetylacetonate: A combined first-principles and reactive molecular dynamics study. Phys. Chem. Chem. Phys..

[27] Karapetyan A., Reymers A., Giorgio S., Fauquet C., Sajti L., Nitsche S., Nersesyan M., Gevorgyan V., Marine W. (2015). Cuprous oxide thin films prepared by thermal oxidation of copper layer. Morphological and optical properties. J. Lumin..

[28] Kobayashi H., Nakamura T., Takahashi N. (2007). Preparation of Cu_2_O films on MgO (1 1 0) substrate by means of halide chemical vapor deposition under atmospheric pressure. Mater. Chem. Phys..

[29] Condorelli G.G., Malandrino G., Fragalà I. (1994). Metal-Organic Chemical Vapor Deposition of Copper-Containing Phases: Kinetics and Reaction Mechanisms. Chem. Mater..

[30] Arana-Chavez D., Toumayan E., Lora F., McCaslin C., Adomaitis R.A. (2010). Modeling the Transport and Reaction Mechanisms of Copper Oxide CVD. Chem. Vap. Depos..

[31] Sekkat A., Nguyen V.H., Masse de La Huerta C.A., Rapenne L., Bellet D., Kaminski-Cachopo A., Chichignoud G., Muñoz-Rojas D. (2021). Open-air printing of Cu_2_O thin films with high hole mobility for semitransparent solar harvesters. Commun. Mater..

[32] Jürgensen L., Höll D., Frank M., Ludwig T., Graf D., Schmidt-Verma A.K., Raauf A., Gessner I., Mathur S. (2020). Controlled growth of Cu and CuO_x_ thin films from subvalent copper precursors. Dalton Trans..

[33] Pousaneh E., Korb M., Dzhagan V., Weber M., Noll J., Mehring M., Zahn D.R.T., Schulz S.E., Lang H. (2018). β-Ketoiminato-based copper(II) complexes as CVD precursors for copper and copper oxide layer formation. Dalton Trans..

[34] Condorelli G.G., Malandrino G., Fragalà I. (1999). Kinetic Study of MOCVD Fabrication of Copper(I) and Copper(II) Oxide Films. Chem. Vap. Depos..

[35] Liu H., Nguyen V.H., Roussel H., Gélard I., Rapenne L., Deschanvres J.-L., Jiménez C., Muñoz-Rojas D. (2019). The Role of Humidity in Tuning the Texture and Electrical Properties of Cu_2_O Thin Films Deposited via Aerosol-Assisted CVD. Adv. Mater. Interfaces.

[36] Barreca D., Gasparotto A., Maccato C., Tondello E., Lebedev O.I., Van Tendeloo G. (2009). CVD of Copper Oxides from a β-Diketonate Diamine Precursor: Tailoring the Nano-Organization. Cryst. Growth Des..

[37] Maruyama T. (1998). Copper oxide thin films prepared by chemical vapor deposition from copper dipivaloylmethanate. Sol. Energy Mater. Sol. Cells.

[38] Lay E., Song Y.-H., Chiu Y.-C., Lin Y.-M., Chi Y. (2005). New CVD Precursors Capable of Depositing Copper Metal under Mixed O2/Ar Atmosphere. Inorg. Chem..

[39] Gupta N., Singh R., Wu F., Narayan J., McMillen C., Alapatt G.F., Poole K.F., Hwu S., Sulejmanovic D., Young M. (2013). Deposition and characterization of nanostructured Cu_2_O thin-film for potential photovoltaic applications. J. Mater. Res..

[40] Smecca E., Valenzano V., Valastro S., Deretzis I., Mannino G., Malandrino G., Accorsi G., Colella S., Rizzo A., La Magna A. (2021). Two-step MAPbI_3_ deposition by Low-Vacuum Proximity Space-Effusion for high-efficiency inverted semitransparent perovskite solar cells. J. Mater. Chem. A.

[41] Smecca E., Jena A.K., Deretzis I., Valastro S., Sanzaro S., Mannino G., Bongiorno C., La Magna A., Miyasaka T., Alberti A. (2021). MAPbI_3_ Deposition by LV-PSE on TiO_2_ for Photovoltaic Application. Front. Electron..

[42] Alberti A., Smecca E., Valastro S., Deretzis J., Mannino G., Bongiorno C., Fisicaro G., La Magna A. (2022). Perovskite Solar Cells from the viewpoint of innovation and sustainability. Phys. Chem. Chem. Phys..

[43] Aithssi A., Atourki L., Labchir N., Ouafi M., Abouabassi K., Elfanaoui A., Ihlal A., Bouabid K. (2020). Optical and dielectric properties of electrochemically deposited p-Cu_2_O films. Mater. Res. Express.

[44] Xue J., Shen Q., Liang W., Liu X., Bian L., Xu B. (2013). Preparation and formation mechanism of smooth and uniform Cu_2_O thin films by electrodeposition method. Surf. Coat. Technol..

[45] Chen A., Long H., Li X., Li Y., Yang G., Lu P. (2009). Controlled growth and characteristics of single-phase Cu_2_O and CuO films by pulsed laser deposition. Vacuum.

[46] Sawicka-Chudy P., Sibinski M., Pawelek R., Wisz G., Cieniek B., Potera P., Szczepan P., Adamiak S., Cholewa M., Glowa L. (2019). Characteristics of TiO_2_, Cu_2_O, and TiO_2_/Cu_2_O thin films for application in PV devices. AIP Adv..

[47] Chua D., Kim S.B., Li K., Gordon R. (2019). Low Temperature Chemical Vapor Deposition of Cuprous Oxide Thin Films Using a Copper(I) Amidinate Precursor. ACS Appl. Energy Mater..

[48] Ben Rabeha M., Khedmia N., Fodhaa M.A., Kanzaria M. (2014). The Effect of Thickness on Optical Band Gap and N-type Conductivity of CuInS_2_ Thin Films Annealed in Air Atmosphere. Energy Procedia.

[49] Sönmezoğlu S., Arslan A., Serin T., Serin N. (2011). The effects of film thickness on the optical properties of TiO_2_–SnO_2_ compound thin films. Phys. Scr..

[50] Zheng W., Chen Y., Peng X., Zhong K., Lin Y., Huang Z. (2018). The Phase Evolution and Physical Properties of Binary Copper Oxide Thin Films Prepared by Reactive Magnetron Sputtering. Materials.

[51] Murali D.S., Kumar S., Choudhary R.J., Wadikar A.D., Jain M.K., Subrahmanyam A. (2015). Synthesis of Cu_2_O from CuO thin films: Optical and electrical properties. AIP Adv..

[52] Schroder D.K. (2006). Semiconductor Material and Device Characterization.

[53] Resende J., Nguyen V.-S., Fleischmann C., Bottiglieri L., Brochen S., Vandervorst W., Favre W., Jiménez C., Deschanvres J.-L., Nguyen N.D. (2021). Grain-boundary segregation of magnesium in doped cuprous oxide and impact on electrical transport properties. Sci. Rep..

[54] Guo Y., Lei H., Xiong L., Li B., Chen Z., Wen J., Yang G., Li G., Fang G. (2017). Single phase, high hole mobility Cu_2_O films as an efficient and robust hole transporting layer for organic solar cells. J. Mater. Chem. A.

[55] Yuan J., Chen Y., Liu X., Xue S. (2021). Dopant-free Hole-transporting Materials for CH_3_NH_3_PbI_3_ Inverted Perovskite Solar Cells with an Approximate Efficiency of 20%. ACS Appl. Energy Mater..

[56] Huang J., Ge C., Qin F., Zou Y., Zhou Y., Li W.-S., Gao X. (2022). Achieve Better Performance of Inverted Perovskite Solar Cells by Using the Fluorinated Polymer as the Electron Transporting Layer. ACS Appl. Energy Mater..

[57] Baikie T., Fang Y., Kadro J.M., Schreyer M., Wei F., Mhaisalkar S.G., Graetzel M., White T.J. (2013). Synthesis and crystal chemistry of the hybrid perovskite (CH_3_NH_3_)PbI_3_ for solid-state sensitized solar cell applications. J. Mater. Chem. A.

[58] Udalova N.N., Nemygina E.M., Zharenova E.A., Tutantsev A.S., Sudakov A.A., Grishko A.Y., Belich N.A., Goodilin E.A., Tarasov A.B. (2020). New Aspects of Copper Electrode Metamorphosis in Perovskite Solar Cells. J. Phys. Chem. C.

