# Supplementary material for: A Low Temperature Growth of Cu2O Thin Films as Hole Transporting Material for Perovskite Solar Cells
Source: Materials (Basel). 2022 Nov 4;15(21):7790. doi: 10.3390/ma15217790 (PMC9657906; doi:10.3390/ma15217790)
Supplement: Supplementary file 1 [file materials-15-07790-s001.zip › materials-1984274-supplementary.pdf]

Electronic Supplementary Material

A Low Temperature Growth of Cu_2O Thin Films as Hole Transporting Material for Perovskite Solar Cells

Anna L. Pellegrino [1,*], Francesca Lo Presti [1], Emanuele Smecca [2,*], Salvatore Valastro [2], Giuseppe Greco [2], Salvatore Di Franco [2], Fabrizio Roccaforte [2], Alessandra Alberti [2,*] and Graziella Malandrino [1,*]

[1] Dipartimento di Scienze Chimiche, Università degli Studi di Catania, INSTM UdR Catania, Viale Andrea Doria 6, 95125 Catania, Italy
[2] National Research Council-Institute for Microelectronics and Microsystems (CNR-IMM), Zona Industriale Strada VIII no. 5, 95121 Catania, Italy
* Correspondence: annalucia.pellegrino@unict.it (A.L.P.); emanuele.smecca@imm.cnr.it (E.S.); alessandra.alberti@imm.cnr.it (A.A.); gmalandrino@unict.it (G.M.)

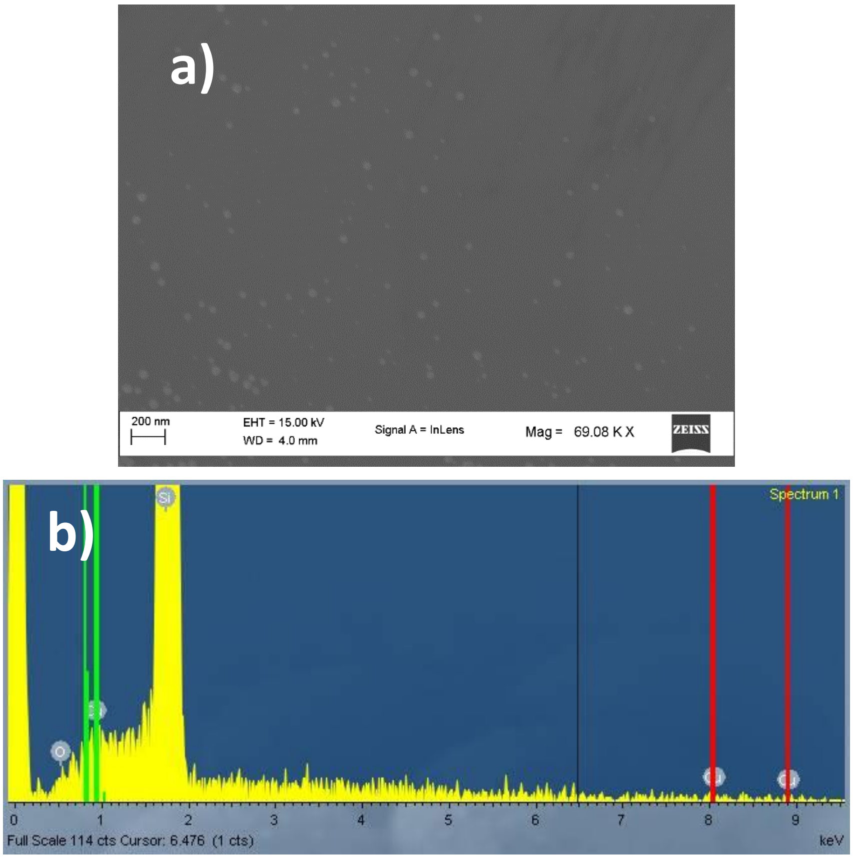

Figure S1. FE-SEM plan-image and EDX spectrum of the Cu_2O thin film deposited on Si substrate at 200 °C.

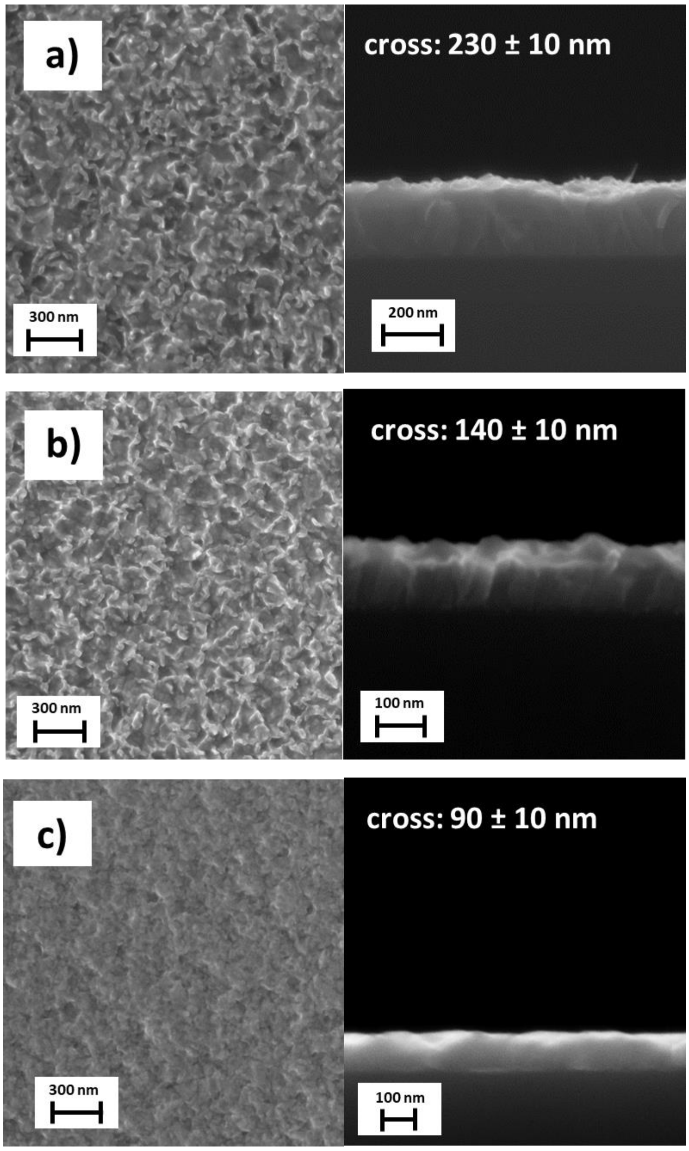

Figure S2. FE-SEM images in plain and cross of Cu_2O thin films deposited on Si substrate at 250 °C with different vaporization temperature and deposition time: a) T_{vap}: 140 °C, t: 30 min; b) T_{vap}: 140 °C, t: 15 min c) T_{vap}: 130 °C t: 15 min.

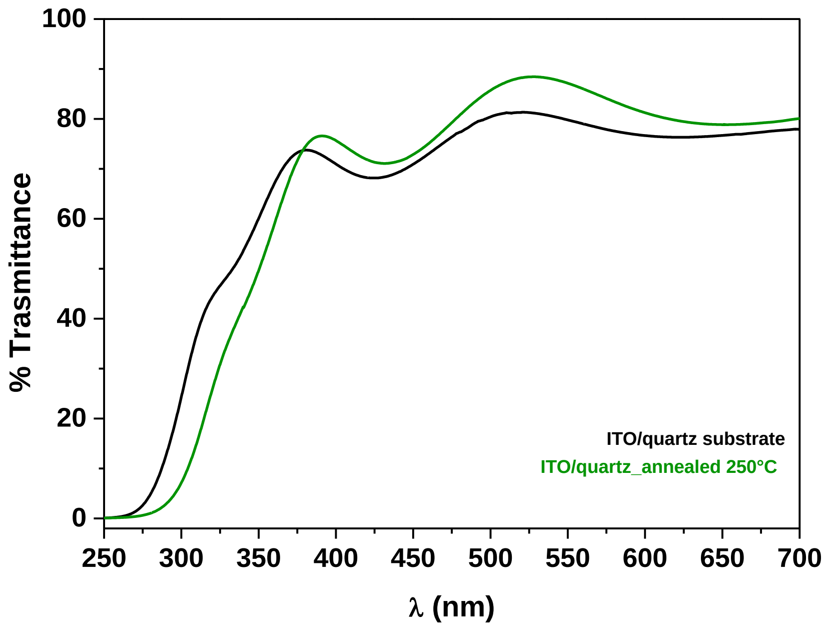

Figure S3. Transmittance spectra of the ITO/quartz substrate (black line); ITO/quartz substrate annealed at 250 °C.

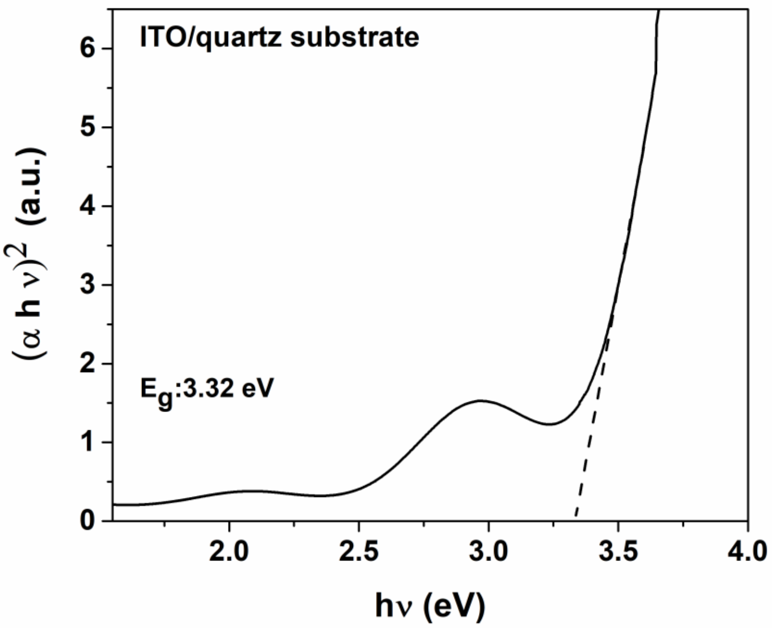

Figure S4. Tauc's plot [$(\alpha h\nu)^2$ against photon energy (hν)] of the ITO/quartz substrate.
